# The effectiveness of an emergency department nursing intervention on psychological symptoms and self-care capacities: A randomized controlled study protocol

**DOI:** 10.1097/MD.0000000000024763

**Published:** 2021-05-28

**Authors:** Xiaoyu Lou, Hua Xu

**Affiliations:** Department of Emergency, Huzhou Central Hospital & Affiliated Central Hospital Huzhou University, Zhejiang, China.

**Keywords:** emergency department, nursing, protocol, revisit

## Abstract

**Background:**

We carried out a randomized trial of an emergency department (ED)-based nursing intervention to evaluate the impact of an ED nursing intervention on ED revisits, patient perceptions of continuity of care, illness perceptions, self-care capacities and psychological symptoms.

**Method:**

We conducted a randomized controlled trial to compare the ED-based intervention with usual care. The protocol was reviewed and approved by the Research Ethics Board of the Huzhou Central Hospital & Affiliated Central Hospital Huzhou University (K901923-021), each participant signed a written consent before participating, and SPIRIT guidelines were followed throughout. To be eligible, patients ready for discharge from the ED had to be at risk for ED return based on 2 criteria: at least one ED visit during the year prior to the initial visit, and current treatment with at least 6 medications. Exclusion criteria included cognitive problems (e.g., dementia) that would preclude provision of informed consent either noted in the medical chart or identified based on the clinical judgment of the project nurse. To avoid multiple interveners for the same patient, we also excluded patients already receiving other regular follow-up (e.g., at a specialized clinic in the hospital or from external resources). The major outcomes were assessed with the Heart Continuity of Care Questionnaire, the Illness Perception Questionnaire-Revised, the Therapeutic Self-Care Tool, the Hospital Anxiety and Depression Scale, and the Self-Reported Medication-Taking Scale.

**Results:**

Two hundred patients who met the inclusion criteria were included in our study, Table 1 showed the effects of nursing intervention on measures of clinical outcomes.

**Discussion:**

The ED is a major entry point into the health care system of many countries. Unnecessary ED revisits may result in overcrowding, increased waiting time, and failure to provide appropriate emergency care. The ED-based interventions literature focuses primarily on service use and ways to reduce ED revisits, with very little focus on impacting secondary outcomes. Because of their potential link with health service utilization, secondary outcomes such as perceived continuity of care, illness perceptions, self-care capacities, psychological symptoms and medication adherence might influence ED revisits. Future research was needed to better understand the complex relationship between ED utilization and a variety of intermediary factors in order to develop interventions that will optimize ED utilization.

## 1 Introduction

Emergency department (ED) revisits are a major concern in health care systems around the world.^[1–3]^ ED revisits contribute to overcrowding, increased waiting times, and impaired quality and safety of care to those in urgent need.^[4,5]^ Extensive empirical evidence documents that most medical EDs serve a relatively small number of frequent users who account for a disproportionately large number of ED visits.^[6]^ Frequent users are typically found to be a socially disadvantaged group with multiple medical and psychiatric disorders and myriad social problems.^[7–9]^ From all perspectives, frequent use of the ED is an undesirable pattern of service use for this vulnerable patient population. Patients receive care that is suboptimal because it is fragmented and episodic, ED health care providers are frustrated by their limited ability to meet frequent users’ many complex needs, and health care systems are burdened by the high costs of excess use of expensive acute services. Shumway et al ^[10]^ found that an intervention delivered by a social worker to frequent emergency department users with psychosocial problems improved secondary outcomes such as peer and social service support, while also reducing emergency department revisits. Two other emergency department-based intervention studies ^[11,12]^ observed some impact on secondary outcomes but did not observe effects on emergency department revisits.

As yet there is no firm evidence about the types of intervention that can reduce emergency room revisits. However, the literature on emergency room revisits suggests patient difficulties with managing their health problems and treatments after discharge may play a role. Based on the controversy, we carried out a randomized trial of an ED-based nursing intervention to evaluate the impact of an ED nursing intervention on ED revisits, patient perceptions of continuity of care, illness perceptions, self-care capacities and psychological symptoms.

## 2 Methods

### 2.1 Design

We conducted a randomized controlled trial to compare the ED-based intervention with usual care. The protocol was reviewed and approved by the Research Ethics Board of the Huzhou Central Hospital &Affiliated Central Hospital Huzhou University (K901923-021), each participant signed a written consent before participating, and SPIRIT guidelines were followed throughout. The study was registered in the public trial registry (researchregistry 6477).

The randomization sequence was generated by an independent statistician using computer. The statistician provided opaque envelopes containing randomization assignments to the project nurse who was blinded to study group until opening the envelope. After the envelope was opened and the patient assigned to the intervention or usual care group, neither the nurse nor the participant were blind to the study group allocation. However, the research assistant who collected outcome measures data by telephone was blinded to study group assignment.

### 2.2 Study setting and participants

The study was conducted in adult patients at the ED of our hospital. To be eligible, patients ready for discharge from the ED had to be at risk for ED return based on 2 criteria: at least 1 ED visit during the year prior to the initial visit, and current treatment with at least 6 medications. Exclusion criteria included cognitive problems (e.g., dementia) that would preclude provision of informed consent either noted in the medical chart or identified based on the clinical judgment of the project nurse. To avoid multiple interveners for the same patient, we also excluded patients already receiving other regular follow-up (e.g., at a specialized clinic in the hospital or from external resources).

Potential participants received usual care from their regular bedside nurse until the ED medical discharge signature was obtained and discharge information was given by the bedside nurse. The study was explained to eligible patients and after they gave informed consent, a self-report questionnaire was administered to collect baseline data before discharge. All patients responded to the sociodemographic questionnaire.

### 2.3 Interventions

In the control group, the project nurse repeated the advice already given by the bedside nurse that patients should contact regular healthcare resources such as telephone health hotlines, family physicians, cardiologists, or emergency services as needed after discharge. No specific intervention was provided to the control group in order to assure that their care was as similar as possible to the usual care in the ED.

We developed the intervention for the experimental group to avoid unscheduled ED revisits, clinical stability should be assured prior to ED discharge, and patients should be prepared to deal with potential postdischarge concerns. For the sake of parsimony and future transfer to clinical practice, and because there was no clear evidence suggesting that a longer intervention would be more powerful than a shorter 1, we developed a short-term intervention that included 3 encounters: one at discharge, and 2 telephone follow-ups at 2 to 4 days and 7 to 10 days postdischarge. The intervention provided by a project nurse was individualized with the potential concerns of each patient assessed using a 19-item clinical disease management tool developed and refined in past studies with similar clients. The assessment evaluated patients’ capacities to cope with:

1. worries about readiness to return home;
2. disease and symptom management;
3. treatment management;
4. activities of daily living and instrumental activities of daily living management;
5. emotions and cognition;
6. informal resources; and
7. the health care system.

For each of the 19 items patients were rated as “no risk”, “presence of risk, but coping strategies in place”, “at risk”, or “not evaluated”. All items had to be evaluated, unless not clinically relevant. When a patient was rated as at-risk for any item, nurses’ interventions included:

1. teaching;
2. normalizing;
3. listening;
4. reassuring;
5. reframing;
6. confronting;
7. providing advice, recommendations;
8. warning;
9. giving positive feedback;
10. referring to external resources; and
11. reinforcing-external resources (e.g., increasing dosage or frequency of resource).

After each encounter, the project nurse checked off which nursing intervention was retained in response to the specific concerns expressed by patients. Because the intervention was individualized, each patient received a different intervention package. Patients were allowed to call the nurse between the planned encounters if they had any questions or concerns. Because the project nurses had access to the hospital chart, they were aware of ED visit characteristics including diagnosis, procedures and treatment, medications, discharge planning, and any other special issues-and could therefore personalize the intervention according to the patient's clinical condition. Four project nurses worked on the project. All project nurses held a bachelor's degree and had at least 5 years of experience in clinical cardiac care, though not necessarily in the ED.

### 2.4 Outcomes measure

The major outcomes were assessed with the Heart Continuity of Care Questionnaire, the Illness Perception Questionnaire-Revised, the Therapeutic Self-Care Tool, the Hospital Anxiety and Depression Scale and the Self-Reported Medication-Taking Scale. In addition to being assessed at baseline in patients who were able to fill out the questionnaires, these measures were readministered by telephone at 30 days postdischarge.

## 3 Results

Two hundred patients who met the inclusion criteria were included in our study, Table 1 showed the effects of nursing intervention on measures of clinical outcomes.

**Table 1:** Effects of nursing intervention on measures of clinical outcomes.

	Experimental group (N = 100)	Control group (N = 100)	*P* value
Revisits			
Heart Continuity of Care Questionnaire Total score Informational continuity subscale Relational continuity subscale Management continuity subscale			
Illness Perception Questionnaire-Revised Consequences of the illness Perceived personal control Perceived treatment control			
Therapeutic Self-Care Tool			
Hospital Anxiety and Depression Scale-Anxiety SubscaleHospital Anxiety and Depression Scale-Deprive symptoms Subscale			
Self-Reported Medication-Taking Scale			

## 4 Discussion

The ED is a major entry point into the health care system of many countries.^[13,12]^ Unnecessary ED revisits may result in overcrowding, increased waiting time, and failure to provide appropriate emergency care.^[14,15]^ The prevalence and persistence of frequent ED use has increased interest in interventions that reduce overuse of the ED by providing patients with more appropriate and consistent medical and social services. A variety of interventions that differ in complexity and intensity have been evaluated in preliminary studies, with promising results.^[16,11,4]^ The ED-based interventions literature focuses primarily on service use and ways to reduce ED revisits, with very little focus on impacting secondary outcomes. Because of their potential link with health service utilization, secondary outcomes such as perceived continuity of care, illness perceptions, self-care capacities, psychological symptoms and medication adherence might influence ED revisits.

This study had several limitations:

1. only patients with cardiovascular disease were included, however, several patients were consulting the ED for non-cardiac problems, this nevertheless limited generalizability of the results;
2. the sample size was based on the primary outcome of ED revisits, and no power analysis was performed for the secondary outcomes examined in the present paper;
3. the most significant threat to validity was the number of patients lost to follow up in the present sample.

These losses were more frequent in the control group than the experimental group. Future research was needed to better understand the complex relationship between ED utilization and a variety of intermediary factors in order to develop interventions that will optimize ED utilization.

## Author contributions

Xiaoyu Lou plans the study design. Hua Xu collects data and reviews the protocol. Xiaoyu Lou writes the manuscript. All authors approve the submission

**Conceptualization:** Xiaoyu Lou.

**Data curation:** Xiaoyu Lou.

**Funding acquisition:** Hua Xu.

**Writing – original draft:** Xiaoyu Lou.

**Writing – review & editing:** Hua Xu.
